# Laboratory Indicators for Identifying Hand, Foot, and Mouth Disease Severity: A Systematic Review and Meta-Analysis

**DOI:** 10.3390/vaccines10111829

**Published:** 2022-10-29

**Authors:** Yaqi Xie, Quanman Hu, Wenjie Jiang, Wangquan Ji, Shuaiyin Chen, Yuefei Jin, Guangcai Duan

**Affiliations:** 1 Department of Epidemiology, College of Public Health, Zhengzhou University, Zhengzhou 450001, China; 2 Henan Key Laboratory of Molecular Medicine, Zhengzhou University, Zhengzhou 450001, China

**Keywords:** hand, foot and mouth disease, laboratory indicators, severity, systematic review, meta-analysis

## Abstract

Objective: The purpose of this study is to study laboratory indicators for the identification of hand, foot, and mouth disease (HFMD) severity. Methods: We searched PubMed, Embase, and the Web of Science for literature that was published before May 2022. The main results are presented as forest plots. Subgroup analyses, sensitivity analyses, and publication bias were also performed. Results: Our study indicated that white blood cells (WBC) (95%CI: 0.205–0.778), blood glucose (95%CI: 0.505–0.778), lymphocytes (95%CI: 0.072–0.239), creatinine (95%CI: 0.024–0.228), interleukin (IL)-2 (95%CI: 0.192–1.642), IL-6 (95%CI: 0.289–0.776), IL-8 (95%CI: 0.499–0.867), IL-10 (95%CI: 0.226–0.930), interferon-γ (IFN-γ) (95%CI: 0.193–2.584), tumor necrosis factor-α (TNF-α) (95%CI: 1.078–2.715), and creatine kinase MB isoenzyme (CK-MB) (95%CI: 0.571–1.459) were associated with an increased risk of HFMD severity, and the results of the sensitivity analysis of these indicators were stable and free of publication bias. Conclusions: Our results suggest that various deleterious immune and metabolic changes can increase the risk of HFMD severity, which can provide a basis for predicting the prognosis and useful evidence for clinicians to manage patients efficiently.

## 1. Introduction

Hand, foot, and mouth disease (HFMD) is a common pediatric disease caused by a variety of human enteroviruses (EVs). HFMD usually affects preschool children (age < 5 years) and has experienced widespread prevalence in this population in recent years [1,2]. The main clinical features of HFMD are fever, mouth ulcers, and skin rashes on the hands, feet, and buttocks [3], and it is usually transmitted through feces, respiratory droplets, contact with blister fluid, or close contact with infected children [4,5]. HFMD is a mild, self-limiting disease, but a number of patients develop severe disease, with central nervous systems (CNS) complications [6]. These CNS complications are brainstem encephalitis, acute flaccid paralysis, neurogenic pulmonary edema, cerebellar ataxia, and aseptic meningitis [7,8,9,10]. Over the past few decades, frequent outbreaks of HFMD have occurred in the Western Pacific [11]. Taiwan experienced a large-scale outbreak of HFMD in 1998, and the distinctive feature of this outbreak was the involvement of the CNS, and nine death cases were reported in Tainan [12]. In 2008 and 2009, enterovirus 71 (EV71) caused 1.26 million cases of HFMD in children and resulted in 377 deaths in mainland China [13]. Since 2009, Japan has reported atypical HFMD cases caused by coxsackievirus-A6 (CV) A6 [14]. Subsequently, a large number of cases and localized outbreaks of HFMD were detected in Vietnam [15] and Thailand [16], as well as in Malaysia [17], and India [18]. As a national project in China, a vaccine against EV71 was developed and introduced to the market in 2016, but widespread pediatric vaccination programs have not yet been introduced in other countries [19]. Although the number of severe cases has declined after the application of EV71 vaccine, the incidence of HFMD remains high and still poses a major threat to children’s health.

The complications and sequelae caused by severe HFMD are very serious, which become a heavy burden on families, communities, and societies. At present, clinicians mainly use clinical manifestations to identify severe cases, which often leads to overdiagnosis. Therefore, it is valuable to seek a laboratory differential diagnostic protocol for severe cases, such as laboratory indicators, to identify HFMD severity at the early stages of the disease. So far, some studies have explored the differences in some laboratory indicators between mild and severe patients. A previous study reported WBC counts and blood glucose levels increased with the severity of HFMD [20]. Growing evidence suggests that inflammatory cytokines play an important role in brainstem encephalitis caused by EV71. Zhang et al. [21] found that the expression of several cytokines and chemokines, such as interferon-γ-inducible protein 10 (IP-10), interleukin (IL)-6, IL-8, granulocyte macrophage-colony stimulating factor (GM-CSF), and monocyte chemoattractant protein-1 (MCP-1), were significantly increased in the plasma of EV71-infected patients compared to healthy individuals. IL-10, IL-13, IL-1β, IL-6, tumor necrosis factor-α (TNF-α), and interferon-γ (IFN-γ) also have been shown in previous studies to predict the occurrence of EV71-associated pulmonary edema [22,23]. Lymphocytes are crucial to regulate immune responses. A previous study found that the imbalance of lymphocyte subsets during EV71 infection, especially the upregulation of Th1/Th2 and Th17/Treg ratios, might be one of the reasons affecting the development of HFMD [24]. Taken together, numerous studies suggest that laboratory indicators are an important way to distinguish HFMD severity, but there exists a lack of comprehensive analysis.

The purpose of this meta-analysis is to investigate the possibility of clinical indicators as predictors of HFMD severity, which can provide a basis for predicting the prognosis and taking early and effective medical treatment.

## 2. Materials and Methods

### 2.1. Diagnosis and Group Classification

According to the Guidelines for Diagnosis and Treatment of HFMD (2018 edition) by the Ministry of Health of the People’s Republic of China (http://www.nhc.gov.cn/cms-search/xxgk/getManuscriptXxgk.htm?id=5db274d8697a41ea84e88eedd8bf8f63, accessed on 31 August 2022), the disease degree of HFMD is classified as follows: (1) the main symptoms are fever, and a rash on the hands, feet, mouth and buttocks. This stage is a common type of HFMD; (2) the manifestations include poor mental health, drowsiness, weakness in sucking, easy startling, headache, vomiting, irritability, limb tremors, muscle weakness, and neck stiffness. Patients who have the above manifestations are severe cases; (3) pre-cardiopulmonary failure occurs within 5 days of disease onset and is characterized by an increased heart rate and respiration, cold sweats, chills at the ends of the extremities, skin flushing, and increased blood pressure. Patients who have the above manifestations are critical cases; (4) the clinical manifestations include tachycardia (individually bradycardia), shortness of breath, cyanosis of the lips and mouth, coughing up pink frothy sputum or bloody fluid, and reduced blood pressure or shock. Patients who have the above manifestations are also critical cases, with a high mortality rate. According to the Chinese guidelines for the diagnosis and treatment of HFMD (2018 edition), stage (1) is defined as mild; stages (2), (3), and (4) are defined as severe.

### 2.2. Literature Search Strategy

A comprehensive search was conducted on PubMed, Embase, and the Web of Science for literature published up until May 2022. The search terms were: Hand, foot, and mouth disease/HFMD, severe/critical, mild, and clinical characters/clinical features/laboratory. The articles selected for this study were published before May 2022 and we searched for relevant reviews and meta-analyses to determine that no similar articles were published. When the abstract of the article met the inclusion criteria, the full text was downloaded for the next screening step.

### 2.3. Inclusion and Exclusion Criteria

The inclusion criteria were: (1) only peer-reviewed, published studies were included, and the subjects were laboratory-confirmed HFMD cases; (2) the study period was specified in the study; (3) the study mentioned the association between severe HFMD and laboratory indicators; (4) the study included clinical symptoms or routine laboratory diagnosis of HFMD (including patients with severe and mild disease groups); (5) the definition of severe and mild cases conformed to the Guidelines for the diagnosis and treatment of HFMD (2018 edition).

The exclusion criteria were as follows: (1) letters, systematic reviews, and meta-analysis; (2) incomplete, questionable, or inconsistent data and information in the articles; (3) the results of laboratory indicators were presented in a manner that was not numerically specific; and (4) animal experiments.

### 2.4. Data Extraction and Quality Assessment

This study was conducted in strict accordance with the PRISMA protocol [25] and the checklist is presented in Supplementary Material Table S1. Two authors independently screened the articles and extracted data from eligible studies using a self-designed standardized data form. Any disagreements regarding quality assessment, data extraction, and data interpretation were resolved through negotiation. All included studies were assessed for quality according to the Newcastle-Ottawa Scale (NOS) [26], with a third party deciding in case of disagreement. A NOS score ≥ 7 was high quality, 5~7 was moderate quality and <5 was low quality. The authors extracted the following data from each article: source journal, authors, study time, the time span of study, sample size, study area, average age, sex ratio, number of cases (control group vs. severe group), the study of pathogens, laboratory indicators, and the NOS score.

### 2.5. Statistical Analysis

For studies with mean ± standard data, a random-effects model/fixed-effects model was used to calculate estimates of standardized mean differences (SMD) and 95% confidence intervals (CI). When the data in a study were not in the form of mean ± standard, we could transform the data from the median (interquartile spacing) provided to estimate the SMD and 95% CI. The I^2^ statistic was used to assess the heterogeneity of included studies, then heterogeneity was shown to be significant if I^2^ was >50% or *p* < 0.05. If the heterogeneity of included studies for an indicator was significant, a random-effects model was used, otherwise, a fixed-effects model was used. A subgroup analysis was used for exploring the potential source of heterogeneity and was based on the year of publication, the time span of study, sample size, average age, sex ratio, and the NOS score. A sensitivity analysis was used to assess the stability of included studies, and the publication bias was assessed using Begg’s test. All statistical analyses were performed using Stata version 11.0 software (Stata Corp, College Station, TX, USA), and the test level was α = 0.05.

## 3. Results

### 3.1. Study Inclusion and Characteristics

**Figure 1:**
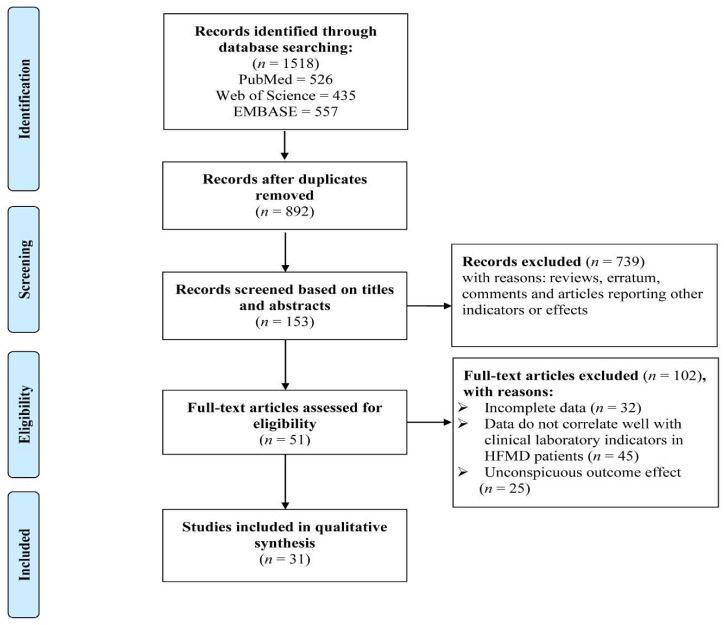
Flow diagram of the study selection process.

A systematic search was conducted on PubMed, the Web of Science, and Embase, and a total of 1518 articles were identified. After removing duplicate articles, 892 records remained. After filtering the titles and abstracts to exclude irrelevant articles, there were still 153 records. After a full-text article assessment of eligibility, a total of 31 papers were finally included in the study, and the search process is shown in Figure 1. In the process of including articles, Cai et al. studied two pathogens, EV71, and CVA16, and the sample sizes of these two serotypes were different, so we included them as two articles. Among them, 21 articles studied EV71, 1 article studied CVA6, 1 article studied CVA16, 6 articles studied EV71 and CVA16, and 2 articles did not identify the pathogenic serotype. All reports of HFMD included in the study were from China. There are 9 high-quality papers and 22 medium-quality papers. The basic information of the included literature is shown in Table 1.

### 3.2. Results of the Meta-Analysis

**Figure 2:**
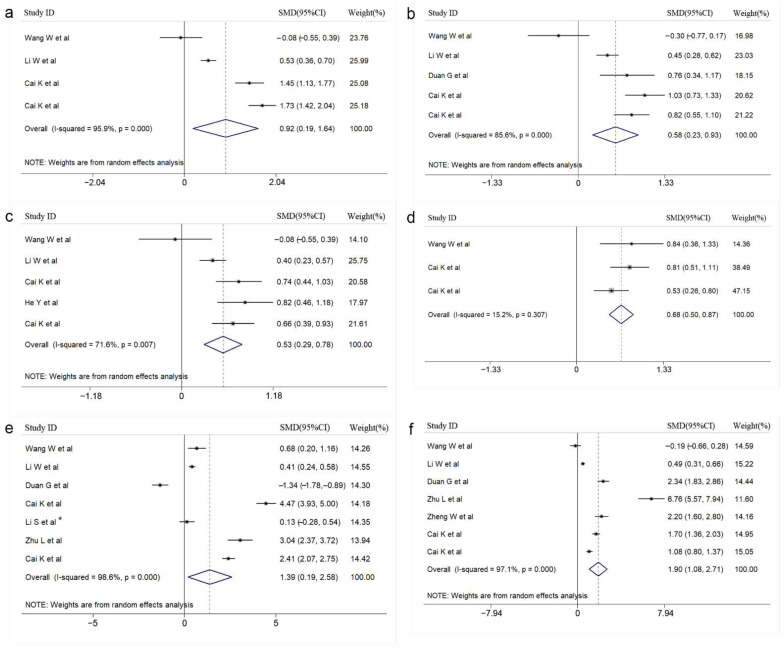
Forest plot for the association of cytokines with HFMD severity (**a**): IL-2; (**b**): IL-10; (**c**): IL-6; (**d**): IL-8; (**e**): IFN-γ; (**f**): TNF-α. (* [24]).

**Figure 3:**
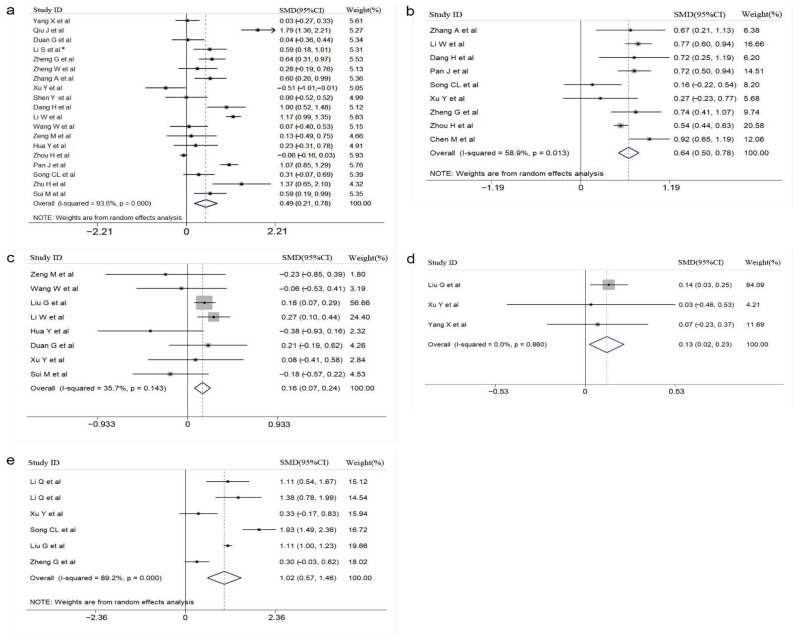
Forest plot for the association of blood indicators with HFMD severity (**a**): WBC; (**b**): blood glucose; (**c**): lymphocytes; (**d**): creatinine; (**e**): CK-MB. (* [24]).

After statistical analysis, 11 meaningful clinical indicators were finally included. The included meaningful indicators can be divided into the following categories, routine laboratory indicators: WBC (SMD = 0.491, 95%CI: 0.205–0.778), blood glucose (SMD = 0.642, 95%CI: 0.505–0.778), lymphocytes (SMD = 0.155, 95%CI: 0.072–0.239), creatinine (SMD = 0.126, 95%CI: 0.024–0.228); cytokine indicators: IL-2 (SMD = 0.917, 95%CI: 0.192–1.642), IL-6 (SMD = 0.533, 95%CI: 0.289–0.776), IL-8 (SMD = 0.683, 95%CI: 0.499–0.867), IL-10 (SMD = 0.578, 95%CI: 0.226–0.930), IFN-γ (SMD = 1.388, 95%CI: 0.193–2.584), TNF-α (SMD = 1.896, 95%CI: 1.078–2.715), and cardiac function index: CK-MB (SMD = 1.015, 95%CI: 0.571–1.459). A forest diagram of each meaningful indicator is shown in Figure 2 and Figure 3. In addition, 12 laboratory indicators were excluded from our statistical analysis. The forest diagram of 12 laboratory indicators that were meaningless after statistical analysis has been placed in Supplementary Material Figures S1 and S2.

### 3.3. Subgroup Analysis

Among the 23 indicators, we conducted a subgroup analysis for indicators with high heterogeneity (I^2^ > 75%) and the number of included articles > 5. Finally, we analyzed the six indicators of IFN-γ, TNF-α, WBC, CK-MB, C-reactive protein (CRP), and alanine aminotransferase (ALT). The results showed no heterogeneity. The relevant results were placed in Supplementary Material Tables S2–S7.

### 3.4. Publication Bias

**Figure 4:**
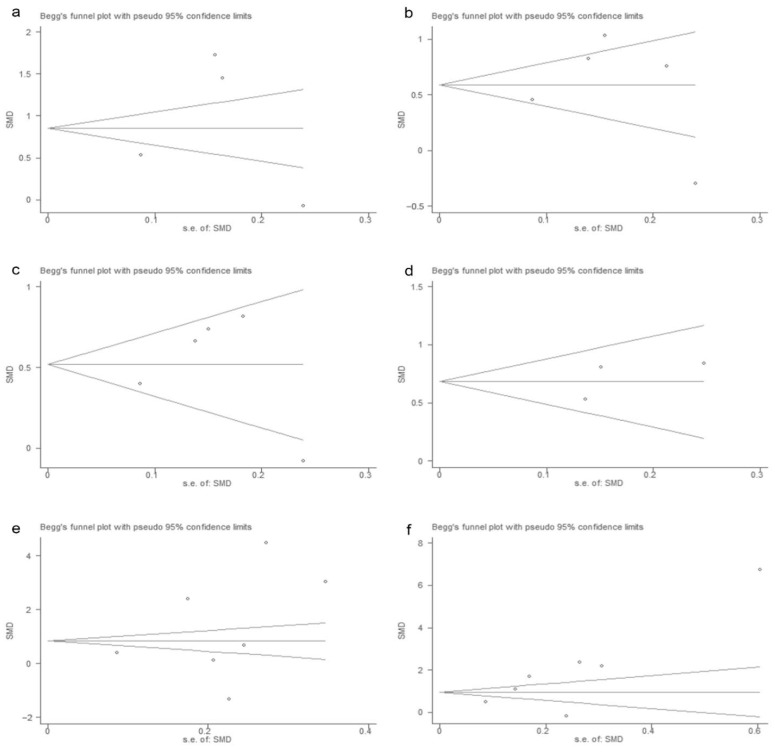
Begg’s funnel plot of laboratory indicators. The main results of the *p*-values of Begg’s test are listed in Table 2 (**a**): IL-2; (**b**): IL-10; (**c**): IL-6 (**d**): IL-8 (**e**): IFN-γ; (**f**): TNF-α.

**Figure 5:**
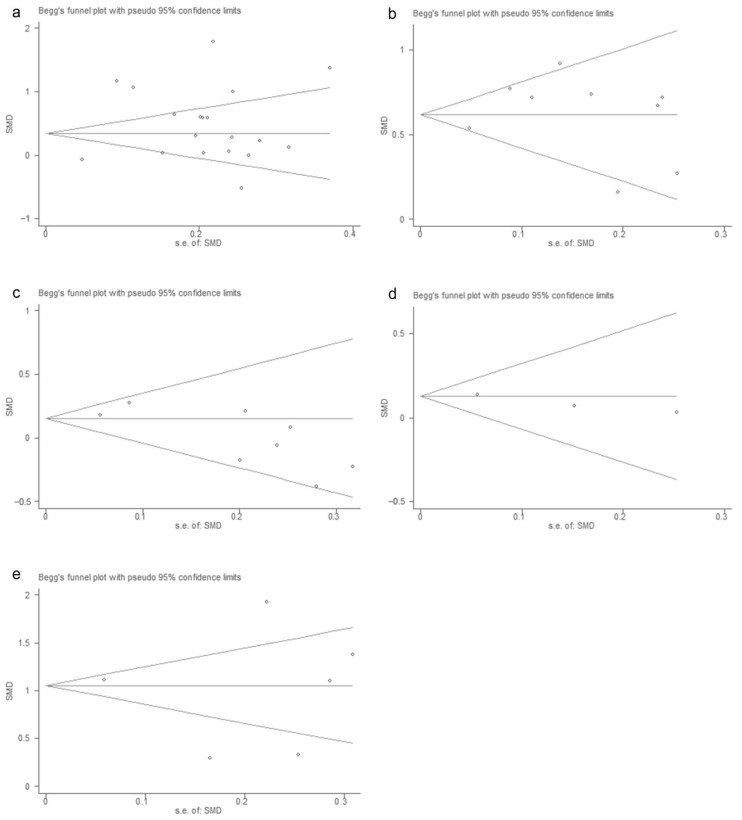
Begg’s funnel plot of laboratory indicators (**a**): WBC; (**b**): blood glucose; (**c**): lymphocytes; (**d**): creatinine; (**e**): CK-MB.

The *p*-values for Begg’s test are shown in Table 2. The results of the Begg’s test are placed in Figure 4 and Figure 5, and the results showed no significant publication bias in the study results for each meaningful indicator. In addition, the publication bias of 12 indicators excluded from the study is shown in Supplementary Material Figures S3 and S4.

### 3.5. Sensitivity Analysis

**Figure 6:**
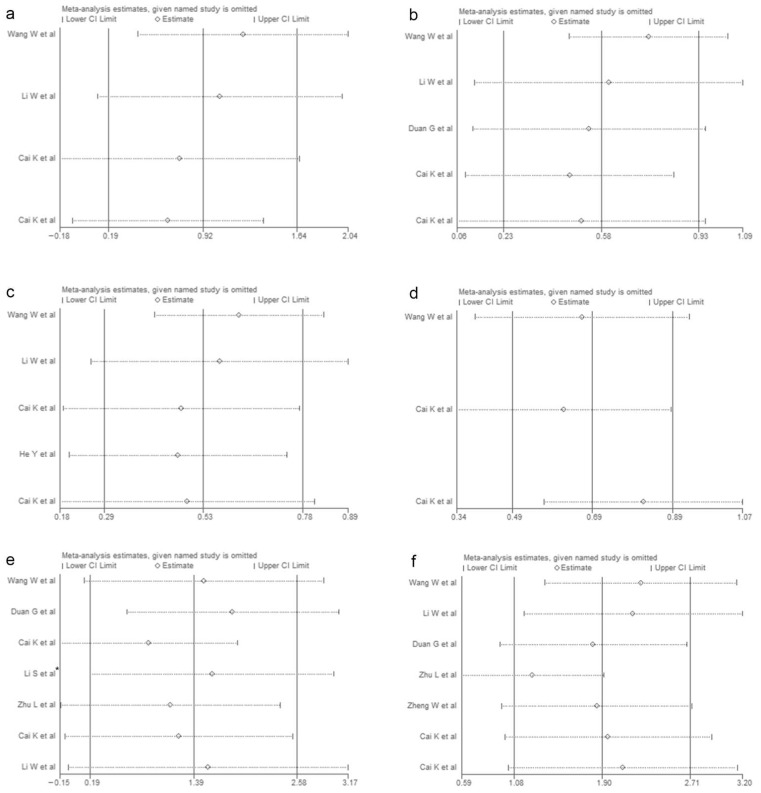
Sensitivity analysis of laboratory indicators (**a**): IL-2; (**b**): IL-10; (**c**): IL-6; (**d**): IL-8; (**e**): IFN-γ; (**f**): TNF-α. (* [24]).

**Figure 7:**
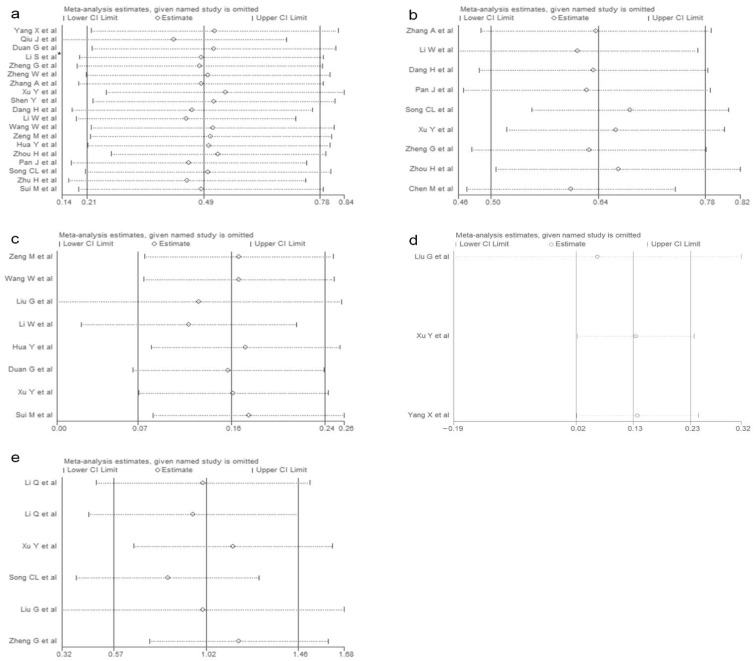
Sensitivity analysis of laboratory indicators (**a**): WBC; (**b**): blood glucose; (**c**): lymphocytes; (**d**): creatinine; (**e**): CK-MB. (* [24]).

A sensitivity analysis was conducted for each indicator, and the results of the sensitivity analysis of the 11 included indicators are shown in Figure 6 and Figure 7. The results of the sensitivity analysis showed that the results of our analysis were robust. In addition, the sensitivity analysis results of 12 indicators excluded from the study are shown in Supplementary Material Figures S5 and S6.

## 4. Discussion

In recent years, the scale and frequency of HFMD outbreaks in the Asia-Pacific region and even in Europe and the United States have become an important public health concern [14]. Severe HFMD has a poor prognosis and may cause serious sequelae, and there are still no effective drugs for the treatment of HFMD. Therefore, it is essential to find candidate indicators that can identify disease severity. When children have HFMD, some laboratory indicators vary with the severity of the disease [27]. In this study, we performed a meta-analysis of laboratory indicators. This is the first systematic review with a meta-analysis conducted to analyze and integrate laboratory indicators of severe HFMD.

First, as reported previously [28], the WBC count and blood glucose increased with the severity of disease, suggesting that these two indicators had certain indications for disease progression. In another study, fifteen patients with a mean blood glucose concentration of 7.66 mmol/L and a WBC count of 11.72 × 10^9^ cells/L recovered after treatment, whereas the other nine patients with a mean blood glucose concentration of 13.84 mmol/L and a WBC count of 16.36 × 10^9^ cells/L had adverse sequelae and even died [29]. A range of studies showed that the WBC count and blood glucose concentration were abnormal laboratory indicators in severe HFMD cases [20,30,31,32].

Cytokines are relatively common indicators of inflammation and there has been a lot of research to discuss the role of cytokines in HFMD pathogenesis. Some independent studies showed a trend toward an increase in inflammatory cytokines and chemokines in severe EV71-infected patients. Overexpression of IL-8, IL-10, IL-2, IL-6, and IFN-γ can exacerbate the inflammatory response and lead to organ dysfunction or dysregulation [21,33]. Shao et al. [34] studied the relationship between plasma cytokines (IL-4, IL-12, IL-18, TNF-α, and IFN-γ) or chemokines (IL-8, RANTES, MCP-1, and IP-10) and HFMD severity. Their results showed that compared with mild patients, the expression levels of cytokines and chemokines were significantly increased in severe patients. Cytokines produced by T helper (Th) cells are of critical importance for the outcome of many infectious diseases. The termed Th1/Th2 classification, although an over-simplification, has proven useful in the analysis of immune responses to viral infections. Li et al. [24] found that Th1 and Tc1 cells produced type 1 cytokines (IL-2, TNF-α and IFN-γ), while Th2 and Tc2 cells secreted type 2 cytokines (IL-4, IL-5 and IL-13) after EV71 infection, and that the percentage of Th1/Tc1 cells and the percentage of Th1/Th2 cells were significantly higher in HFMD patients compared to healthy individuals. In addition, Li et al. [35] observed a positive correlation between IL-6 and IFN-γ concentrations in the cerebrospinal fluid of patients with EV71-associated meningitis, and Mcloughlin et al. [36] also reported that IFN-γ can regulate IL-6 signaling through sIL-6R, suggesting that EV71-induced IFN-γ secretion may enhance IL-6 production. The above data reveal that EV71 can disrupt Th1/Th2 balance as pathogenic factors in the development of HFMD through cytokine imbalances induced by EV71. The immune system is a sophisticated and complex network, whose dynamic equilibrium sustains health. Viral infection describes the interaction between the action of viral invasion and the reaction of the body’s inflammatory defensive response [37]. Overexpression of inflammatory chemokines and cytokines can induce excessive immune responses and cause cytokine cascades and cytokine storms through autocrine and paracrine mechanisms, leading to severe systemic inflammation and organ dysfunction [38,39]. Taken together, cytokines/chemokines are involved in the pathogenesis of HFMD and have potential value in monitoring disease progression and predicting prognosis.

There were some previous studies on the role of lymphocytes in disease progression. Zhao et al. [40] found that the percentages of T lymphocyte subsets and B lymphocyte populations in the peripheral blood of HFMD patients changed significantly, suggesting that cellular immune responses were involved in the pathogenesis of HFMD. T lymphocytes play an important role in cellular immunity and can be divided into (cluster of differentiation 8) CD8+ T cells (including Tc1, Tc2, and Tc17), and CD4+ T cells (including Th1, Th2, Th17, and Treg subsets). Th17 is involved in the pathological process of EV71 infection [41]. Tc17 and Th17 cells are featured in the production of IL-17. IL-17A is the main effector molecule of Th17 cells, by stimulating the production of cytokines and chemokines, such as CXCL5, CXCL6/IL-6, and CXCL8/IL-8, and then promoting neutrophil differentiation, maturation, and migration, thereby enhancing the inflammatory response [42]. This evidence suggests that a cascade of Th17 response may be involved in the disease progression of HFMD. In addition, Th1/Th2 cell imbalance and Th17/Treg imbalance may trigger HFMD severity and play an important role in disease progression. A previous study showed that CD4+ and CD8+ T lymphocytes were related to disease progression and adverse outcomes [43]. IL-10 is known to be produced by Th1/Th2 cells, macrophages, dendritic cells (DC), B cells, and various subsets of CD4+and CD8+ T cells [44,45]. IL-10 limits the production of pro-inflammatory cytokines (IL-6, IL-12, IL-18, and TNF-α) and chemokines (IL-8, IP-10, MCP-1, and RANTES) through its direct action on monocytes and macrophages [46]. Similarly, IL-10 can act directly on CD4+ T cells and inhibit the production of IL-2, IFN-γ, and IL-4 [47]. IL-10 directly regulates intrinsic and adaptive Th1 and Th2 responses by limiting T cell activation and differentiation in lymph nodes and suppressing pro-inflammatory responses in tissues and participates in further immune processes [40,48,49]. Jun et al. [50] indicated that T cell receptor (TCR) and innate receptor co-stimulation provided a T-cell-intrinsic signal which generated a dramatic, synergistic cytokine response dominated by IL-10. In previous studies, by analyzing lymphocytes from children with HFMD, it was found that the increased frequency of B cells in the blood might reflect a severe stage of HFMD, and an excessive immune response might increase the severity of HFMD [51]. Together, focusing on lymphocytes as a laboratory marker of HFMD severity makes sense for clinical practice.

CK-MB has been shown in several studies to be associated with the occurrence of severe illness or death in HFMD patients [52]. However, we did not find out the mechanism of this change in severe patients. In addition, we also did not find any mechanism for the change in creatinine in severe HFMD patients. Therefore, further study is required in the future to investigate the possible mechanisms. Yi et al. [53] found that several routine laboratory indicators and clinical features were risk factors for HFMD severity. However, the authors only discussed four laboratory indicators, which were IL-6, CD4+, neutrophils, and lymphocytes. We think the advantage of this meta-analysis is the relatively large number of articles included and the more comprehensive discussion of laboratory indicators.

Regarding the included laboratory indicators, some issues need to be clarified. We included a total of 23 indicators for analysis and finally included 11 statistically significant indicators. The 12 excluded indexes were aspartate aminotransferase (AST), ALT, CRP, hemoglobin, neutrophils, platelets, lactate dehydrogenase (LDH), creatine kinase (CK), interleukin-4 (IL-4), monocytes, immunoglobulin A(IgA), and immunoglobulin G (IgG). These 12 indicators cannot accurately reflect the disease progression of HFMD. Unfortunately, there are few studies on the mechanisms of these indicators. Therefore, this reminds us that these results should be explored in the future

However, some limitations of this study are worth noting. Firstly, the source of heterogeneity of some indicators was not found by the subgroup analysis. Secondly, we focused on laboratory indicators, while we did not pay too much attention to clinical symptoms, risk factors, pathogens, and other indicators. Despite these limitations, this study provides data and a basis for further research. Our study helped to identify indicators that can predict the severity of HFMD and understanding these indicators will provide a reasonable explanation for the development of interventions and treatment for severe HFMD.

## 5. Conclusions

Our results suggest that various deleterious immune and metabolic changes can increase the risk of HFMD severity, which will provide useful evidence for clinicians to manage patients efficiently.

## Figures and Tables

**Table 1:** Basic characteristics of the included studies.

Literature Number	Author	Time Span	Sample Size	Sex Ratio	Average Age	Pathogen	NOS
1	Zhang A et al.	2014.4–2015.3	83	1.19	2.23	EVA71	6
2	Zeng M et al.	2010.7–2010.9	40	4	2.77	EVA71	8
3	Wang W et al.	2012.5–2012.7	84	1.8	2.66	EVA71	5
4	Qiu J et al.	2012.1–2014.12	362	1.74	1.75	EVA71, CVA16	6
5	Liu G et al.	2012.3–2015.7	2532	2.1	2	EVA71	7
6	Liu C et al.	2012.1–2015.12	717	1.73	2.5	EVA71	5
7	Li W et al.	2012.3–2012.10	571	1.59	2.58	EVA71	7
8	Li Q et al.	2018.1–2018.12	53	3.08	2.67	EVA71	7
9	Hua Y et al.	2014–2015	52	1.6	2.8	EVA71	6
10	Duan G et al.	2012.4–2012.11	102	1.55	1.93	EVA71	7
11	Dang HX et al.	2015.6–2016.9	89	NR	2.32	EVA71	8
12	Cai K et al.	2014.1–2016.12	190	NR	NR	EVA71	6
13	Cai K et al.	2014.1–2016.12	234	NR	NR	CVA16	6
14	Dang H et al.	2015.6–2018.3	111	1.27	2.5	EVA71, CVA16	7
15	He Y et al.	2012–2014	132	1.93	2.85	EVA71	7
16	Li Q et al.	2017.6–2018.6	58	3.46	2.63	EVA71	7
17	Pan J et al.	2008–2009	369	1.88	4.17	EVA71, CVA16	5
18	Song CL et al.	2010.5–2012.9	164	1.88	NR	EVA71, CVA16	6
19	Xu Y et al.	2012.4–2013.9	107	1.49	2.33	EVA71	5
20	Yang X et al.	2017.1–2017.12	261	1.43	1.69	CVA6	6
21	Zheng G et al.	2009.1–2016.12	179	1.67	2.04	EVA71	5
22	Zhou H et al.	2008.5–2011.11	2379	1.39	3.2	EVA71, CVA16	6
23	Shen Y et al.	2017.8–2018.3	66	1.64	2.49	EVA71	6
24	Yang T et al.	2010.1–2011.6	356	1.87	2.11	EVA71	6
25	Li S et al.	2011.5–2011.8	95	1.32	2.02	EVA71	6
26	Zhu L et al.	2013.1–2016.1	76	1.53	4.64	EVA71	6
27	Zheng W et al.	2014.7–2015.7	70	0.79	2.18	EVA71	6
28	Zhu H et al.	2018.3	39	1.29	2	NR	6
29	Han F et al.	2013.7–2015.7	300	1.38	2.7	EVA71, CVA16	5
30	Chen M et al.	2013.1–2014.6	240	1.06	1.85	EVA71	5
31	Sui M et al.	2013.4–2013.6	100	1.86	1.49	NR	6

NR: not reported.

**Table 2:** Begg’s test results of the final inclusion indicators.

Clinical Indicators	Number of Literature	Begg’s Test (*p* Value)
IL-2	4	1.000
IL-10	4	0.734
IL-6	5	0.806
IL-8	3	1.000
IFN-γ	7	0.368
TNF-α	7	0.072
WBC	19	0.576
Blood glucose	9	0.466
Lymphocytes	8	0.108
Creatinine	3	1.000
CK-MB	7	1.000

## Data Availability

Not applicable.

## Supplementary Materials

The following are available online at https://www.mdpi.com/article/10.3390/vaccines10111829/s1, Table S1: PRISMA checklist; Table S2: Subgroup analysis results of IFN-γ; Table S3: Subgroup analysis results of TNF-α; Table S4: Subgroup analysis results of WBC; Table S5: Subgroup analysis results of ALT; Table S6: Subgroup analysis results of CK-MB; Table S7: Subgroup analysis results of CRP; Figure S1: Forest plot without indicators of research significance; Figure S2: Forest plot without indicators of research significance; Figure S3: Begg’s test results without indicators of research significance; Figure S4: Begg’s test results without indicators of research significance; Figure S5: Sensitivity analysis results without indicators of the study significance; Figure S6: Sensitivity analysis results without indicators of the study significance.
