# Supplementary material for: Laboratory Indicators for Identifying Hand, Foot, and Mouth Disease Severity: A Systematic Review and Meta-Analysis
Source: Vaccines (Basel). 2022 Oct 29;10(11):1829. doi: 10.3390/vaccines10111829 (PMC9694715; doi:10.3390/vaccines10111829)
Supplement: Supplementary file 1 [file vaccines-10-01829-s001.zip › vaccines-1949849-supplementary.pdf]

Supplemental table S1. PRISMA checklist

Section and Topic	Item #	Checklist item	Location where item is reported
**TITLE**			
Title	1	Identify the report as a systematic review.	1
**ABSTRACT**			
Abstract	2	See the PRISMA 2020 for Abstracts checklist.	2
**INTRODUCTION**			
Rationale	3	Describe the rationale for the review in the context of existing knowledge.	3
Objectives	4	Provide an explicit statement of the objective(s) or question(s) the review addresses.	3
**METHODS**			
Eligibility criteria	5	Specify the inclusion and exclusion criteria for the review and how studies were grouped for the syntheses.	5
Information sources	6	Specify all databases, registers, websites, organisations, reference lists and other sources searched or consulted to identify studies. Specify the date when each source was last searched or consulted.	5
Search strategy	7	Present the full search strategies for all databases, registers and websites, including any filters and limits used.	5
Selection process	8	Specify the methods used to decide whether a study met the inclusion criteria of the review, including how many reviewers screened each record and each report retrieved, whether they worked independently, and if applicable, details of automation tools used in the process.	5
Data collection process	9	Specify the methods used to collect data from reports, including how many reviewers collected data from each report, whether they worked independently, any processes for obtaining or confirming data from study investigators, and if applicable, details of automation tools used in the process.	5
Data items	10a	List and define all outcomes for which data were sought. Specify whether all results that were compatible with each outcome domain in each	5

Section and Topic	Item #	Checklist item	Location where item is reported
		study were sought (e.g. for all measures, time points, analyses), and if not, the methods used to decide which results to collect.	
	10b	List and define all other variables for which data were sought (e.g. participant and intervention characteristics, funding sources). Describe any assumptions made about any missing or unclear information.	6
Study risk of bias assessment	11	Specify the methods used to assess risk of bias in the included studies, including details of the tool(s) used, how many reviewers assessed each study and whether they worked independently, and if applicable, details of automation tools used in the process.	6
Effect measures	12	Specify for each outcome the effect measure(s) (e.g. risk ratio, mean difference) used in the synthesis or presentation of results.	6
Synthesis methods	13a	Describe the processes used to decide which studies were eligible for each synthesis (e.g. tabulating the study intervention characteristics and comparing against the planned groups for each synthesis (item #5)).	5
	13b	Describe any methods required to prepare the data for presentation or synthesis, such as handling of missing summary statistics, or data conversions.	6
	13c	Describe any methods used to tabulate or visually display results of individual studies and syntheses.	6
	13d	Describe any methods used to synthesize results and provide a rationale for the choice(s). If meta-analysis was performed, describe the model(s), method(s) to identify the presence and extent of statistical heterogeneity, and software package(s) used.	6
	13e	Describe any methods used to explore possible causes of heterogeneity among study results (e.g. subgroup analysis, meta-regression).	6
	13f	Describe any sensitivity analyses conducted to assess robustness of the synthesized results.	6
Reporting bias assessment	14	Describe any methods used to assess risk of bias due to missing results in a synthesis (arising from reporting biases).	6
Certainty assessment	15	Describe any methods used to assess certainty (or confidence) in the body of evidence for an outcome.	6

Section and Topic	Item #	Checklist item	Location where item is reported
**RESULTS**			
Study selection	16a	Describe the results of the search and selection process, from the number of records identified in the search to the number of studies included in the review, ideally using a flow diagram.	6 and Fig.1
	16b	Cite studies that might appear to meet the inclusion criteria, but which were excluded, and explain why they were excluded.	6
Study characteristics	17	Cite each included study and present its characteristics.	6 and Table1
Risk of bias in studies	18	Present assessments of risk of bias for each included study.	7 and Fig.4-5, Table 2
Results of individual studies	19	For all outcomes, present, for each study: (a) summary statistics for each group (where appropriate) and (b) an effect estimate and its precision (e.g. confidence/credible interval), ideally using structured tables or plots.	7 and Fig.2-3
Results of syntheses	20a	For each synthesis, briefly summarise the characteristics and risk of bias among contributing studies.	7
	20b	Present results of all statistical syntheses conducted. If meta-analysis was done, present for each the summary estimate and its precision (e.g. confidence/credible interval) and measures of statistical heterogeneity. If comparing groups, describe the direction of the effect.	7
	20c	Present results of all investigations of possible causes of heterogeneity among study results.	7
	20d	Present results of all sensitivity analyses conducted to assess the robustness of the synthesized results.	7 and Fig.6-7
Reporting biases	21	Present assessments of risk of bias due to missing results (arising from reporting biases) for each synthesis assessed.	7
Certainty of evidence	22	Present assessments of certainty (or confidence) in the body of evidence for each outcome assessed.	7

Section and Topic	Item #	Checklist item	Location where item is reported
**DISCUSSION**			
Discussion	23a	Provide a general interpretation of the results in the context of other evidence.	8
	23b	Discuss any limitations of the evidence included in the review.	9
	23c	Discuss any limitations of the review processes used.	9
	23d	Discuss implications of the results for practice, policy, and future research.	9
**OTHER INFORMATION**			
Registration and protocol	24a	Provide registration information for the review, including register name and registration number, or state that the review was not registered.	NA
	24b	Indicate where the review protocol can be accessed, or state that a protocol was not prepared.	NA
	24c	Describe and explain any amendments to information provided at registration or in the protocol.	NA
Support	25	Describe sources of financial or non-financial support for the review, and the role of the funders or sponsors in the review.	11
Competing interests	26	Declare any competing interests of review authors.	11
Availability of data, code and other materials	27	Report which of the following are publicly available and where they can be found: template data collection forms; data extracted from included studies; data used for all analyses; analytic code; any other materials used in the review.	11

Supplemental table S2. Subgroup analysis results of IFN-γ

**IFN-γ**		**pooled effects**	**95%CI-LCI**	**95%CI-UCI**	**I^2**	***P* value**
Time	≤2014	-0.024	-0.781	0.733	94.6%	< 0.001
	>2014	3.229	1.988	4.611	95.1%	< 0.001
Time span	≤5	0.388	-0.148	0.925	65.9%	0.087
	5 < ≤10	-0.452	-2.162	1.259	98.1%	< 0.001
	> 10	3.299	1.988	4.611	95.1%	< 0.001
Sample size	≤100	1.261	-0.243	2.765	96.2%	< 0.001
	100 < ≤200	1.480	-0.407	3.367	99.2%	< 0.001
Male-female sex ratio	≤1.55	0.446	0.263	0.629	7.3%	0.299
	> 1.55	0.596	-1.535	2.728	98.2%	< 0.001
	NR	3.427	1.410	5.443	97.5%	< 0.001
Average age	≤2.5	-0.601	-2.038	0.837	95.6%	< 0.001
	2.5 < ≤5	1.338	0.068	2.607	96.3%	< 0.001
	NR	3.427	1.410	5.443	97.5%	< 0.001
NOS	≤6	2.137	0.650	3.624	98.1%	< 0.001
	> 6	1.388	0.193	2.584	98.1%	< 0.001

Abbreviation: IFN-γ, interferon-γ; CI, confidence interval; NR, not reported

Supplemental table S3. Subgroup analysis results of TNF-α

TNF-α		pooled effects	95%CI-LCI	95%CI-UCI	I^2	*P* value
Time	≤2015	0.868	-0.285	2.022	96.4%	< 0.001
	2015 < ≤2018	4.447	-0.019	8.914	97.8%	< 0.001
	>2018	1.384	0.783	1.985	86.9%	< 0.001
Time span	≤20	1.191	0.129	2.253	96.4%	< 0.001
	> 20	2.987	1.388	4.586	97.7%	< 0.001
Sample size	≤100	2.872	-0.247	5.990	98.4%	< 0.001
	100 < ≤200	1.989	1.358	2.620	76.5%	< 0.001
	> 200	0.774	0.187	1.360	92.1%	< 0.001
Male-female sex ratio	≤1.55	3.679	1.671	5.687	96.0%	< 0.001
	1.55 < ≤2	0.185	-0.470	0.841	85.7%	0.008
	NR	1.384	0.783	1.985	86.9%	0.006
Average age	≤2.5	2.282	1.890	2.674	0.00%	0.718
	2.5 < ≤5	2.210	0.152	4.268	98.3%	< 0.001
	NR	1.384	0.783	1.985	86.9%	0.006
NOS	≤6	2.164	0.996	3.332	97.1%	< 0.001
	> 6	1.398	-0.424	3.220	97.8%	< 0.001

Abbreviation: TNF-α, tumor necrosis factor-α; CI, confidence interval; NR, not reported

Supplemental table S4. Subgroup analysis results of WBC

**WBC**		**pooled effects**	**95%-LCI**	**95%-UCI**	**I^2**	***P* value**
Time	≤2013	0.438	-0.256	1.132	96.6%	< 0.001
	2013 < ≤2015	0.454	-0.069	0.976	91.3%	< 0.001
	2015 < ≤2017	0.718	0.191	1.245	87.0%	< 0.001
	>2017	0.345	-0.241	0.931	86.9%	< 0.001
Time span	≤10	0.394	-0.044	0.832	88.8%	< 0.001
	10 < ≤20	0.301	-0.190	0.792	90.4%	< 0.001
	> 20	0.719	0.061	1.377	95.9%	< 0.001
	NR	1.373	0.648	2.098	NR	NR
Sample size	≤70	0.358	-0.044	0.761	60.2%	0.040
	70 < ≤100	0.484	0.245	0.722	23.1%	0.272
	100 < ≤200	0.304	-0.145	0.754	83.2%	< 0.001
	> 200	0.788	0.094	1.483	98.3%	< 0.001
Male-female sex ratio	≤1.3	0.759	0.350	1.168	62.6%	0.046
	1.3 < ≤1.5	0.020	-0.284	0.323	76.6%	0.005
	1.5 < ≤1.7	0.444	-0.083	0.971	90.8%	< 0.001
	> 1.7	0.678	0.189	1.167	89.4%	< 0.001
Average age	≤2	0.742	0.048	1.435	92.8%	< 0.001
	2 < ≤2.5	0.389	0.052	0.725	75.9%	< 0.001
	> 2.5	0.450	-0.153	1.053	97.4%	< 0.001
	NR	0.310	-0.073	0.694	NR	NR
NOS	≤5	0.344	-0.308	0.966	92.5%	< 0.001
	5 < ≤6	0.497	0.165	0.830	90.3%	< 0.001
	> 6	0.491	0.205	0.778	90.6%	< 0.001

Abbreviation: WBC, white blood cells; CI, confidence interval; NR, not reported

Supplemental table S5. Subgroup analysis results of ALT

**ALT**		**pooled effects**	**95%-LCI**	**95%-UCI**	**I^2**	***P* value**
Time	⩽2015	0.093	-0.054	0.240	0.00%	0.702
	>2015	0.416	-0.664	1.496	97.6%	< 0.001
Time span	≤10	0.093	-0.054	0.240	0.00%	0.702
	10 < ⩽20	-0.198	-0.762	0.366	73.8%	0.051
	> 20	1.050	-0.659	2.759	98.5%	< 0.001
Sample size	⩽200	-0.041	-0.330	0.249	49.1%	0.117
	> 200	0.263	-0.330	0.885	98.5%	< 0.001
Male-female sex ratio	⩽1.5	0.263	-0.330	0.855	98.3%	< 0.001
	> 1.5	0.107	-0.027	0.241	0.00%	0.820
Average age	⩽2	0.080	-0.158	0.317	0.00%	0.793
	2 < ⩽2.5	-0.147	-0.832	0.538	81.2%	0.021
	> 2.5	0.650	-0.641	1.942	98.5%	< 0.001
NOS	⩽5	0.379	-0.797	1.554	97.5%	< 0.001
	> 5	0.101	-0.036	0.239	0.00%	0.943

Abbreviation: ALT, alanine aminotransferase; CI, confidence interval

Supplemental table S6. Subgroup analysis results of CK-MB

CK-MB		pooled effects	95%-LCI	95%-UCI	I^2	*P* value
Time	≤2017	1.015	0.571	1.459	94.6%	< 0.001
	>2017	0.922	0.287	1.557	74.7%	0.019
Time span	≤15	1.233	0.821	1.644	0.00%	0.513
	15 < ≤30	1.134	-0.428	2.696	95.5%	< 0.001
	> 30	0.719	-0.076	1.514	95.3%	< 0.001
Sample size	≤150	0.922	0.287	1.557	74.7%	0.019
	> 150	1.015	0.571	1.459	94.6%	< 0.001
Male-female sex ratio	≤2	0.850	-0.195	1.896	94.8%	< 0.001
	> 2	1.119	1.008	1.230	0.00%	0.690
Average age	≤2.3	0.719	-0.076	1.514	95.3%	< 0.001
	> 2.3	0.922	0.287	1.557	74.7%	0.019
	NR	1.926	1.491	2.362	NR	NR
NOS	≤6	0.850	-0.195	1.896	94.8%	< 0.001
	> 6	1.119	1.008	1.230	0.00%	0.690

Abbreviation: CK-MB, creatine kinase MB isoenzyme; CI, confidence interval; NR, not reported

Supplemental table S7. Subgroup analysis results of CRP

**CRP**		**pooled effects**	**95%-LCI**	**95%-UCI**	**I^2**	***P* value**
Time	≤2014	0.114	-0.026	0.253	2%	0.382
	>2014	0.638	-0.232	1.507	98.1%	< 0.001
Time span	≤10	0.098	-0.114	0.311	32.8%	0.226
	> 10	1.166	-0.801	3.133	98.7%	< 0.001
	NR	0.706	0.032	1.380	NR	NR
Sample size	≤150	0.638	-0.232	1.507	50.0%	0.111
	> 150	1.103	-0.715	2.921	99.2%	< 0.001
Male-female sex ratio	≤1.5	1.093	-0.445	2.632	97.9%	< 0.001
	> 1.5	0.088	-0.056	0.232	0.00%	0.377
Average age	≤2	0.221	-0.640	1.083	79.4%	0.028
	2 < ≤2.5	0.308	0.003	0.614	0.00%	0.928
	> 2.5	1.570	-1.280	4.420	99.6%	< 0.001
	NR	0.167	-0.216	0.549	NR	NR
NOS	≤6	1.060	-0.486	2.607	98.2%	< 0.001
	> 6	0.088	-0.106	0.282	20.9%	0.283

Abbreviation: CRP, C-reactive protein; CI, confidence interval; NR, not reported

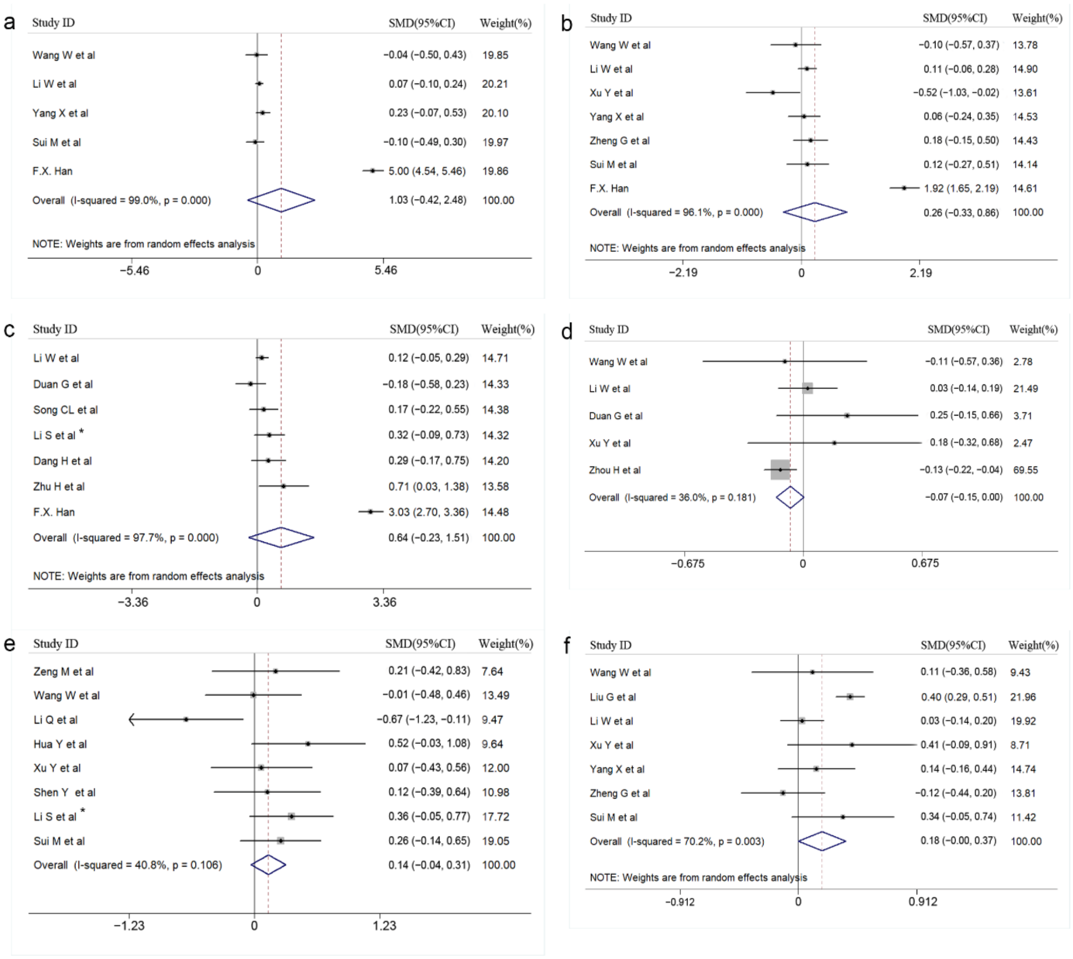

Supplemental figure S1 Forest plot without indicators of research significance (a): AST; (b): ALT; (c): CRP; (d): HB; (e): neutrophils; (f): PLT. (* : [24] Li, S.; Cai, C.; Feng, J.; Li, X.; Wang, Y.; Yang, J.; Chen, Z. Peripheral T lymphocyte subset imbalances in children with enterovirus 71-induced hand, foot and mouth disease. Virus Res. 2014, 180, 84–91.)

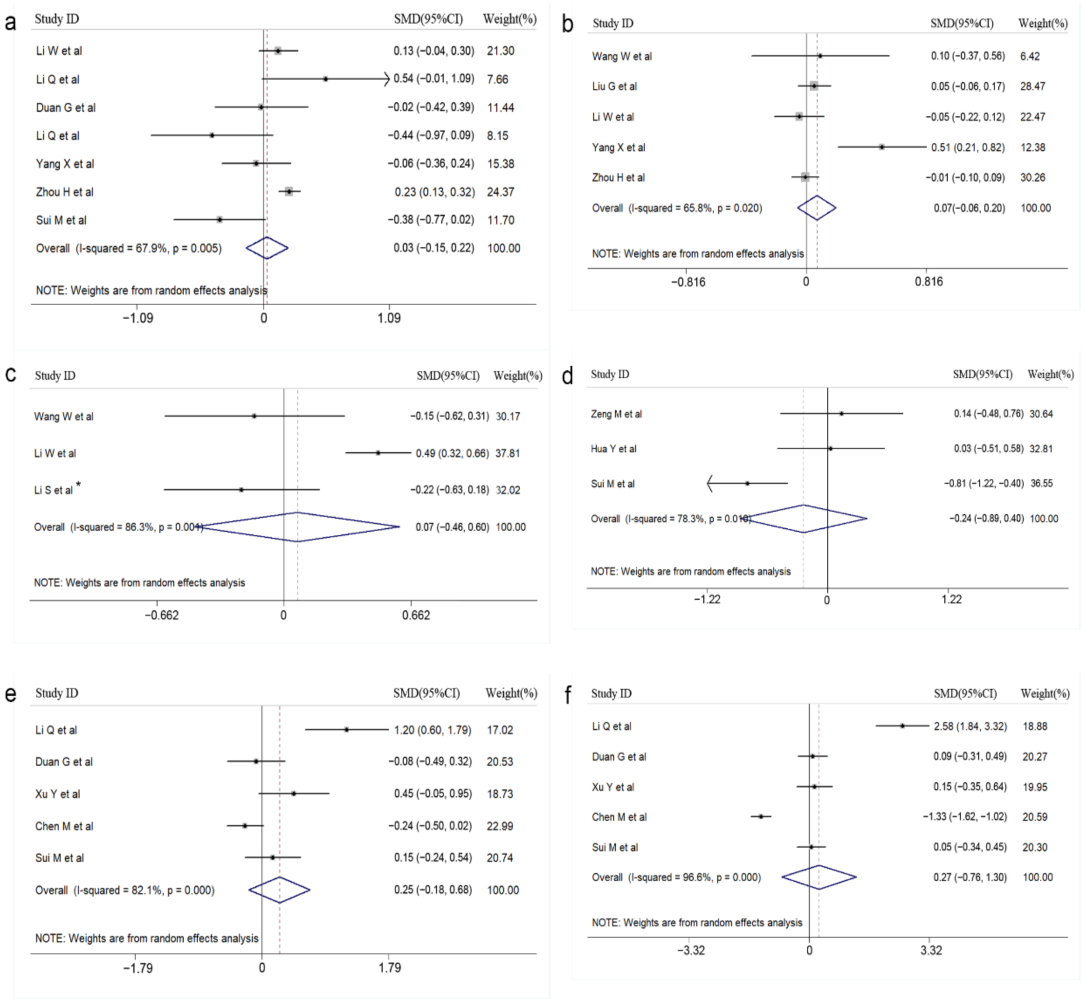

Supplemental figure S2 Forest plot without indicators of research significance (a): LDH; (b): CK; (c): IL-4; (d): monocytes; (e): IgA; (f): IgG. (* : [24] Li, S.; Cai, C.; Feng, J.; Li, X.; Wang, Y.; Yang, J.; Chen, Z. Peripheral T lymphocyte subset imbalances in children with enterovirus 71-induced hand, foot and mouth disease. Virus Res. 2014, 180, 84–91.)

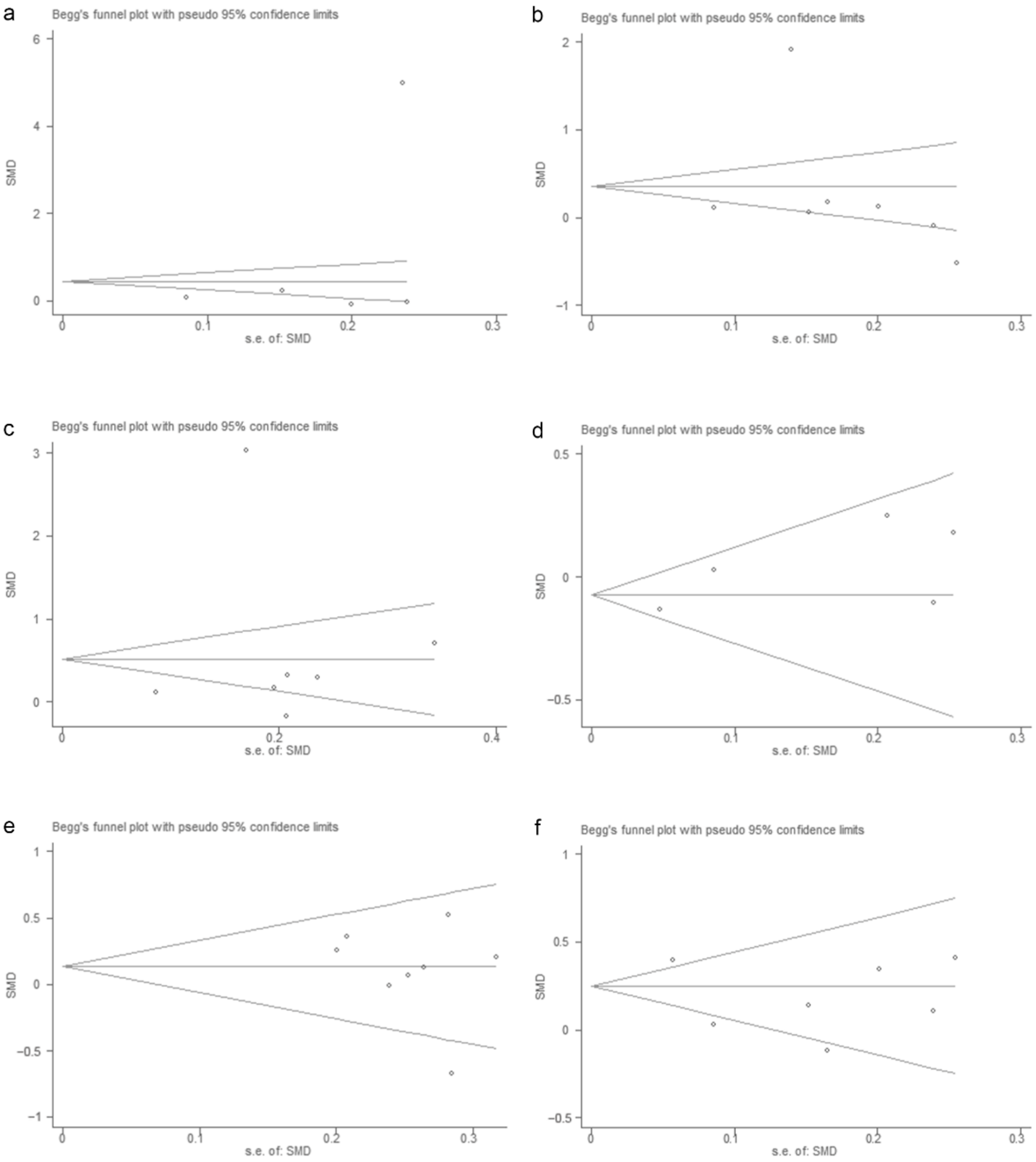

Supplemental figure S3 Begg's test results without indicators of research significance (a): AST; (b): ALT; (c): CRP; (d): HB; (e): neutrophils; (f): PLT.

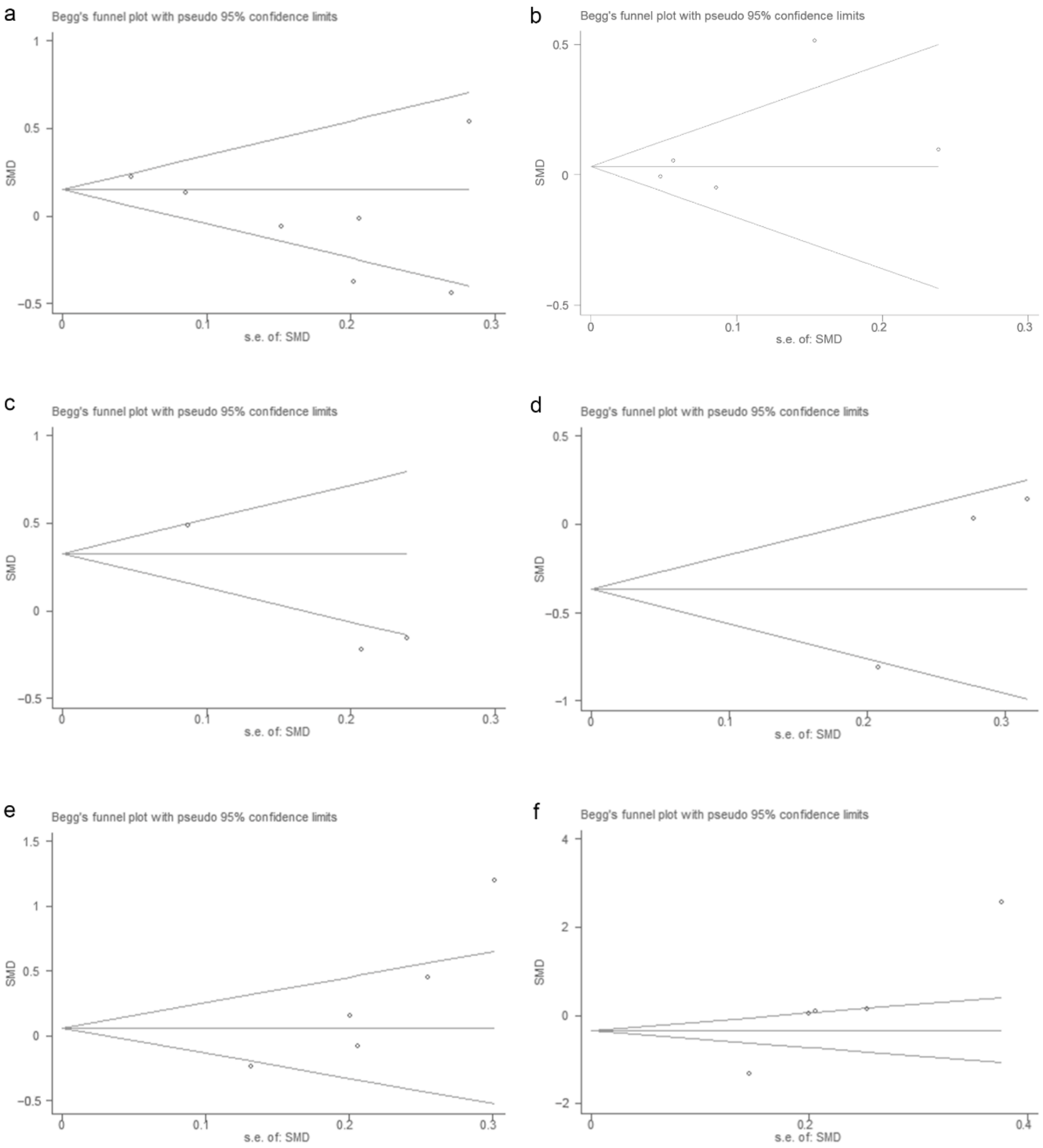

Supplemental figure S4 Begg's test results without indicators of research significance (a): LDH; (b): CK; (c): IL-4; (d): monocytes; (e): IgA; (f): IgG.

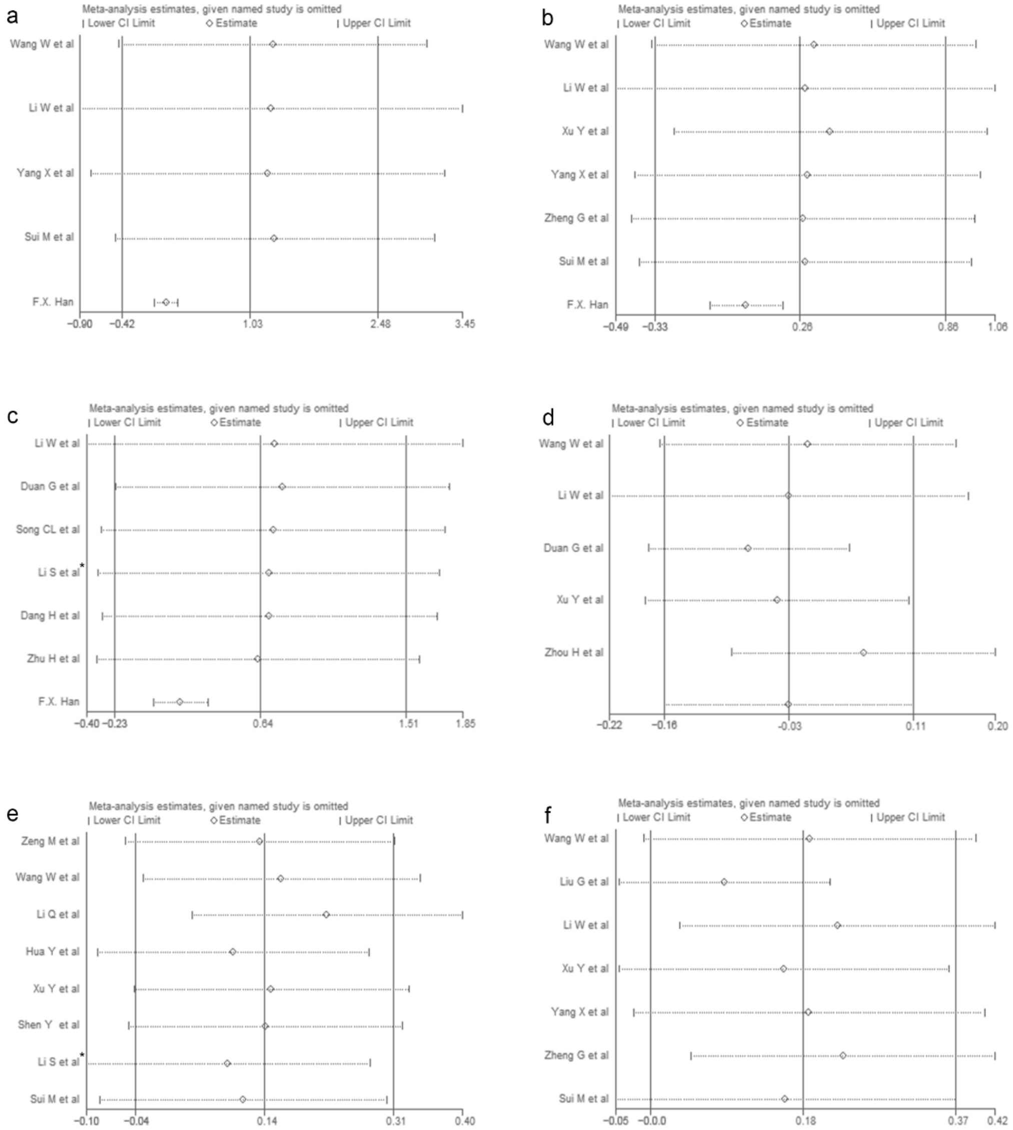

Supplemental figure S5 Sensitivity analysis results without indicators of the study significance (a): AST; (b): ALT; (c): CRP; (d): HB; (e): neutrophils; (f): PLT. (* : [24] Li, S.; Cai, C.; Feng, J.; Li, X.; Wang, Y.; Yang, J.; Chen, Z. Peripheral T lymphocyte subset imbalances in children with enterovirus 71-induced hand, foot and mouth disease. Virus Res. 2014, 180, 84–91.)

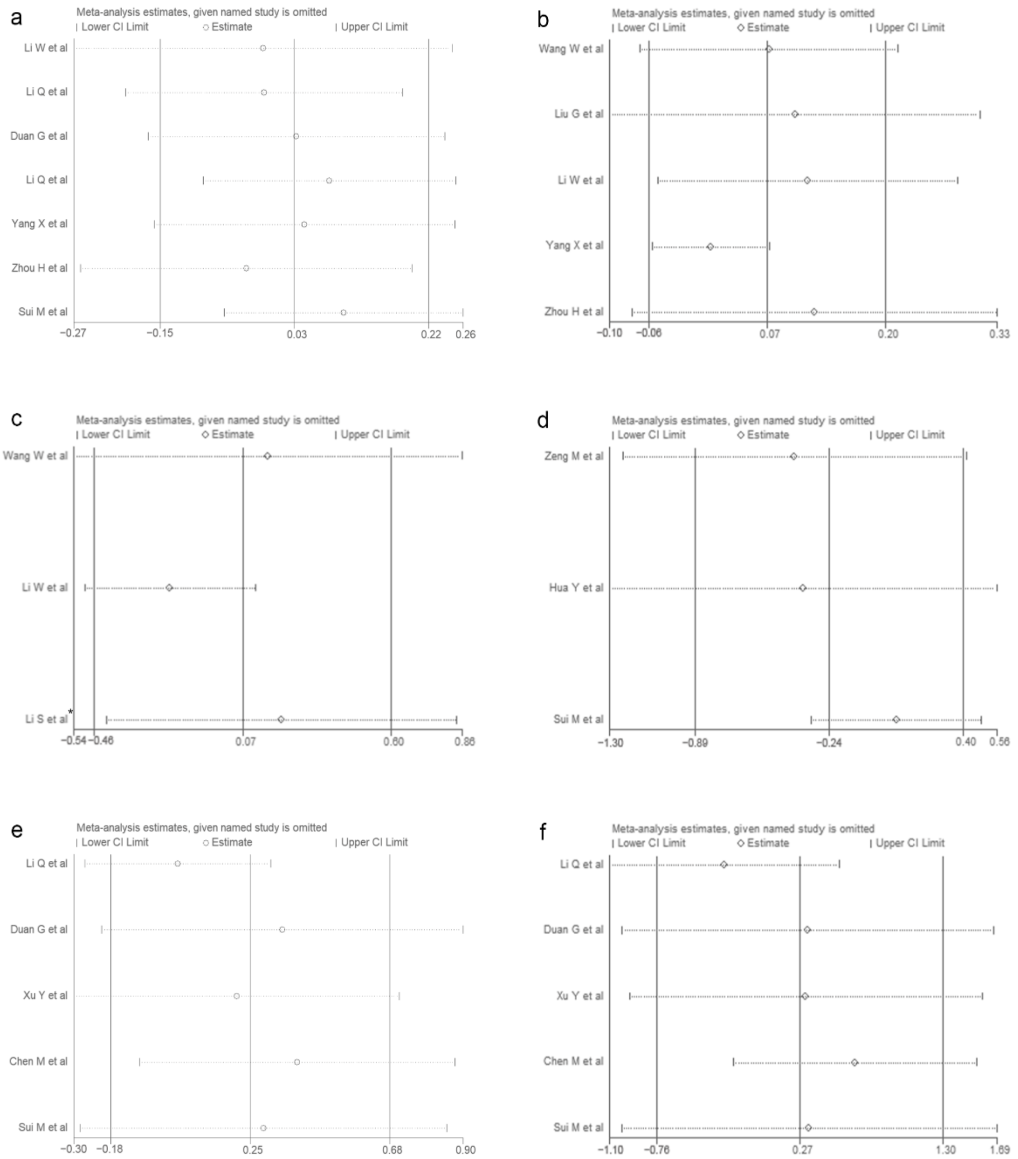

Supplemental figure S6 Sensitivity analysis results without indicators of the study significance (a): LDH; (b): CK; (c): IL-4; (d): monocytes; (e): IgA; (f): IgG. (* : [24] Li, S.; Cai, C.; Feng, J.; Li, X.; Wang, Y.; Yang, J.; Chen, Z. Peripheral T lymphocyte subset imbalances in children with enterovirus 71-induced hand, foot and mouth disease. Virus Res. 2014, 180, 84–91.)
